# Vascular endothelial growth factor modified macrophages transdifferentiate into endothelial-like cells and decrease foam cell formation

**DOI:** 10.1042/BSR20170002

**Published:** 2017-06-21

**Authors:** Dan Yan, Yujuan He, Jun Dai, Lili Yang, Xiaoyan Wang, Qiurong Ruan

**Affiliations:** 1 Department of Pathology, Medical College, Wuhan University of Science and Technology, Wuhan, Hubei, P.R. China; 2 Department of Pathology, Affiliated Tianyou Hospital of Wuhan University of Science and Technology, Wuhan, Hubei, P.R. China; 3 Institute of Pathology of Tongji Hospital, Tongji Medical College, Huazhong University of Science and Technology, Wuhan, Hubei, P.R. China

**Keywords:** endothelial-like cells, foam cell, macrophages, vascular endothelial growth factor

## Abstract

Macrophages are largely involved in the whole process of atherosclerosis from an initiation lesion to an advanced lesion. Endothelial disruption is the initial step and macrophage-derived foam cells are the hallmark of atherosclerosis. Promotion of vascular integrity and inhibition of foam cell formation are two important strategies for preventing atherosclerosis. How can we inhibit even the reverse negative role of macrophages in atherosclerosis? The present study was performed to investigate if overexpressing endogenous human vascular endothelial growth factor (VEGF) could facilitate transdifferentiation of macrophages into endothelial-like cells (ELCs) and inhibit foam cell formation. We demonstrated that VEGF-modified macrophages which stably overexpressed human VEGF (hVEGF_165_) displayed a high capability to alter their phenotype and function into ELCs *in vitro*. Exogenous VEGF could not replace endogenous VEGF to induce the transdifferentiation of macrophages into ELCs *in vitro*. We further showed that VEGF-modified macrophages significantly decreased cytoplasmic lipid accumulation after treatment with oxidized LDL (ox-LDL). Moreover, down-regulation of CD36 expression in these cells was probably one of the mechanisms of reduction in foam cell formation. Our results provided the *in vitro* proof of VEGF-modified macrophages as atheroprotective therapeutic cells by both promotion of vascular repair and inhibition of foam cell formation.

## Introduction

Atherosclerosis is the primary cause of mortality and morbidity in cardiovascular disease, which is the leading cause of deaths in industrialized society [1,2]. It is generally recognized that endothelial dysfunction or disruption is the initial process of atherosclerosis [3–5]. Endothelial progenitor cells (EPCs) are capable of facilitating re-endothelialization through direct differentiation into endothelial cells and/or via the paracrine mechanisms [6,7]. Moreover, a recent research points that endothelial to mesenchymal transition (EndMT) contributes to atherosclerotic pathobiology and is associated with complex plaques that may be related to clinical events [8]. Therefore, maintenance of the endothelial homeostasis and integrity by promoting early repair is an important strategy for preventing atherosclerosis [9,10].

Monocytes are recruited from peripheral blood and attach to the activated/damaged endothelium, then migrate to subendothelial space and differentiate into macrophages. The uptake of oxidized LDL (ox-LDL) by monocyte-derived macrophage induces foam cell formation, which is a hallmark of the development of atherosclerosis [11,12]. Inhibition of foam cell formation in early stage is another fascinating approach for the prevention of progression of atherosclerosis.

Cells of the monocyte-macrophage lineage are characterized by considerable diversity and plasticity [13]. Monocytes are not only the precursors of lipid-laden foam cell macrophages, but also display high developmental plasticity to differentiate under appropriate stimulation into different cell types, including endothelial lineage cells [14]. Can we take the advantage of the potential developmental relationship between monocytes/macrophages and endothelial lineage cells to alter some properties of macrophages to inhibit even the reverse negative role of macrophages in atherosclerosis?

In our previous research, we transiently transfected mouse primary macrophages with human vascular endothelial growth factor 165 (hVEGF_165_) plasmid and found that they could transdifferentiate into endothelial-like cells (ELCs) and incorporated in the newly formed vessel [15,16]. It indicated that the macrophages overexpressing VEGF might have a promising role like EPCs for cell-based therapy. However, the primary macrophages transiently transfected with hVEGF_165_ only expressed the target protein for a few days. To further investigate the effect of stable endogenous VEGF on macrophages, we have successfully established hVEGF_165_-ZsGreen1-RAW264.7 cells – a mouse macrophage cell line stably overexpressing hVEGF_165_ by lentiviral vector [17].

Then, the aims of the present study were to: (i) identify the phenotype and function of hVEGF_165_-ZsGreen1-RAW264.7 cells to determine whether they transdifferentiate into ELCs; (ii) investigate the capability of hVEGF_165_-ZsGreen1-RAW264.7 cells to become foam cells and the underlying mechanism.

## Materials and methods

### Cell culture

RAW264.7 macrophages stably overexpressing hVEGF named as hVEGF_165_- ZsGreen1-RAW264.7 in the present paper was the already established cell line in our laboratory [17]. Briefly, hVEGF_165_ was subcloned into the lentiviral expression vector pLVX-IRES-ZsGreen1 to construct recombinant pLVX-hVEGF_165_-IRES-ZsGreen1. RAW264.7 cells were infected with this recombined lentiviral vector containing hVEGF_165_ and ZsGreen1, which were then purified by FACS twice according to GFP ZsGreen1. Then, we obtained the mouse macrophage cell line stably overexpressing hVEGF_165_ after verification by fluorescent microscopy, PCR, Western blot and ELISA. hVEGF_165_- ZsGreen1-RAW264.7 cells were cultured in Dulbecco’s modified Eagle’s medium (DMEM; Invitrogen Life Technologies, Carlsbad, CA, U.S.A.) high glucose containing 20% (v/v) FBS (Lonza, Walkersville, MD, U.S.A.) and 10 ng/ml basic fibroblast growth factor (bFGF; Invitrogen). RAW 264.7 macrophages only transfected with ZsGreen1 were named as ZsGreen1-RAW264.7 and untransfected RAW264.7 (ATCC, Manassas, VA, U.S.A.) were both used as controls. There is another group of untransfected RAW264.7 with treatment of exogenous recombinant hVEGF_165_ protein (PeproTech Inc, Rocky Hill, NJ, U.S.A.) in 50 ng/ml for 48 h named as VEGF-treated RAW264.7.

### qRT-PCR

To detect the level of some endothelial marker genes, mRNA expression of VEGF receptor-2 fetal liver kinase 1 (FLK-1), von Willebrand factor (vWF), endothelial NO synthase (eNOS), vascular endothelial-cadherin (VE-cadherin) and Tie-2 were evaluated by qRT-PCR using the THUNDERBIRD SYBR qPCR Mix (Toyobo, Japan) with gene-specific primers on a 7500 Fast Real-Time PCR system (Applied Biosystems, Alameda, CA, U.S.A.). The expression of *CD36* mRNA in ox-LDL-induced hVEGF_165_-ZsGreen1-RAW264.7 cells was also detected as above. Specific fragments were amplified and β-actin was also amplified to serve as an internal standard. The results were normalized to β-actin and presented as fold difference relative to RAW267.4 control. All primer sequences are shown in Supplementary Table S1. All experiments were repeated three times, and the representative data are shown.

### Western blot analysis

To detect the level of some endothelial marker proteins, expression of FLK-1, vWF and eNOS were performed by Western blot analysis. Briefly, cells were lysed in RIPA buffer (Sigma–Aldrich, MO, U.S.A.), followed by protein quantitation using Pierce BCA Protein Assay Kit (Thermo Fisher Scientific, Pittsburgh, PA, U.S.A.). Cell lysates containing the same amount of proteins were separated on SDS/PAGE, followed by transferring on to the nitrocellulose membranes. After non-specific blocking with 5% non-fat milk in TBS containing 0.05% Tween 20 at room temperature for 1 h, the membranes were incubated with the specific antibodies toward mouse FLK-1 (Invitrogen; 1:1000), vWF (Santa Cruz Biotechnology, Santa Cruz, CA, U.S.A.; 1:600) and eNOS (Abcam, Cambridge, MA, U.S.A.; 1:500) at 4°C overnight. Subsequently, membranes were incubated with appropriate secondary antibody, and protein bands were visualized using ECL (Thermo Fisher). Bands were quantitated by densitometry and normalized to those of β-actin (Abcam; 1:1000). The expression of CD36 protein in ox-LDL-induced hVEGF_165_-ZsGreen1-RAW264.7 cells was detected by the specific antibody (Abcam; 1:1000) as above. All experiments were repeated three times and the representative data are shown.

### *In vitro* angiogenesis assay

The formation of tubular-like structures was assessed using Matrigel (BD Biosciences, San Jose, CA, U.S.A) by *in vitro* angiogenesis assay. A 96-well plate was precoated with Matrigel and incubated at 37°C for 2 h prior to the addition of 2 × 10^4^ cells/well suspended in 100 µl conditioned medium. Following additional incubation for 24 h, three fields were chosen at random and the formation of tubular-like structures was observed using an inverted microscope (IX51; Olympus, Japan).

### ELISA for VEGF concentration

After 72 h of culture, VEGF concentration in supernatants was measured in triplicate using the VEGF human ELISA kit and VEGF mouse ELISA kit (both from Abcam), respectively. Briefly, VEGF standards and samples were pipetted into wells and VEGF present in a sample was bound to the wells by the immobilized antibody. The wells were washed and biotinylated anti-human or anti-mouse VEGF antibody was added. After washing away unbound biotinylated antibody, peroxidase (HRP)–conjugated streptavidin was pipetted into the wells. The wells were again washed, a TMB substrate solution was added to the wells and color developed in proportion to the amount of VEGF bound. The Stop Solution changed the color from blue to yellow, and the intensity of the color was measured at 450 nm. VEGF concentrations were calculated (in pg/ml) with the standard curve.

### Foam cell formation assay

An *in vitro* foam cell formation assay was performed as described previously with minor modification [18]. Briefly, hVEGF_165-_ZsGreen1-RAW264.7, ZsGreen1-RAW264.7, or RAW264.7 were cultured in 12-well plate in serum-free medium and treated with 100 μg/ml ox-LDL (Yiyuan Biotechnologies, Guangzhou, China) for 24 h to induce foam cell formation. Oil red O powder (Sigma–Aldrich, MO, U.S.A.) was dissolved in isopropanol (0.5%; Sigma–Aldrich). The stock was then diluted to 0.3% oil red O solution with distilled H_2_O and filtered through a 0.22-μm filter. After fixation with 4% paraformaldehyde for 1 h at room temperature, cells were stained with oil red O solution to detect the lipid accumulation for 5 min. Then, the cells were observed with a microscope and those containing oil red O - positive fat droplets were considered foam cells.

### Quantitation of total lipid content

Quantitation of lipid accumulation in cells was measured based on a previously published protocol [18]. Cells stained with oil red O were treated with 1 ml of 60% isopropanol for 1 h to redissolve the oil red O and absorbance was detected at 518 nm through a spectrophotometer.

### Statistical analysis

Data are presented as means ± S.D. The statistical significance of differences between groups was analyzed using one-way ANOVA with Tukey’s test post hoc. Values of *P*<0.05 were considered significant.

## Results

### Stable overexpression of VEGF induces macrophages to acquire phenotypic characteristics of ELCs

**Figure 1:**
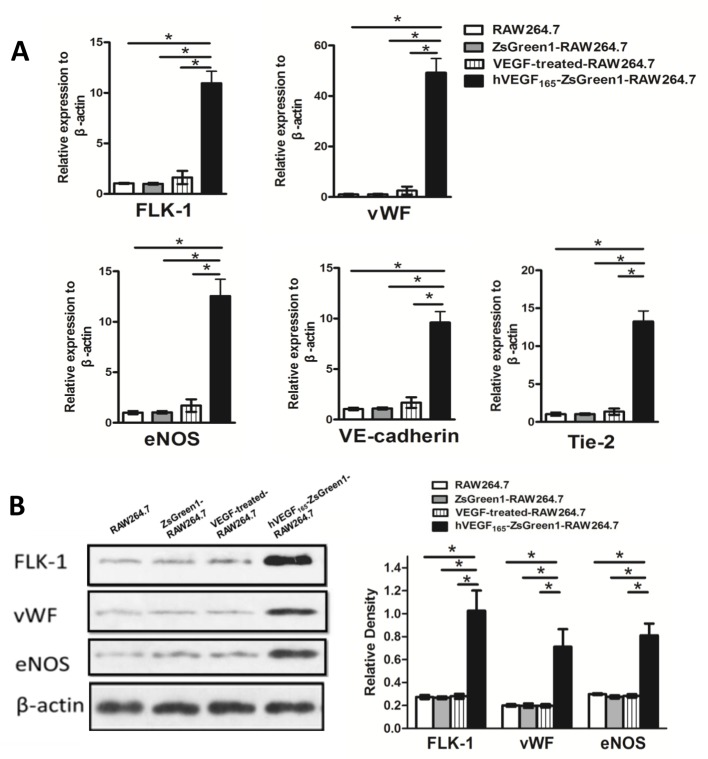
Effect of stable overexpression of VEGF on macrophages in phenotypic characteristics (**A**) Quantitative analysis of mRNA expression of endothelial markers by real-time PCR. mRNA expression of FLK-1, vWF, eNOS, VE-cadherin, and Tie-2 were dramatically increased in hVEGF_165_-ZsGreen1-RAW264.7 cells compared with untransfected RAW264.7, ZsGreen1-RAW264.7, and exogenous VEGF-treated RAW264.7. Data are normalized to β-actin and presented as fold difference relative to RAW267.4 control. (**B**) Western blot analysis of endothelial markers expression. Protein expression of FLK-1, vWF, and eNOS were dramatically increased in hVEGF_165_-ZsGreen1-RAW264.7 cells compared with untransfected RAW264.7, ZsGreen1-RAW264.7, and exogenous VEGF-treated RAW264.7. All images are representatives of three independent experiments each performed in triplicate, and graphs depict the value of mean and S.D**.** * indicates *P*<0.01.

Compared with ZsGreen1-RAW264.7, untransfected RAW264.7 or VEGF-treated-RAW264.7 cells, all of which are small and round in shape, hVEGF_165_-ZsGreen1-RAW264.7 cells appeared elongated spindle-like shape like endothelial cells (Supplementary Figure S1). To investigate the impact of autocrine VEGF on macrophages, the expression of endothelial cell markers in hVEGF_165_-ZsGreen1-RAW264.7 were investigated by qRT-PCR and Western blot. ZsGreen1-RAW264.7 or untransfected RAW264.7 cells were used as controls. Meanwhile, the VEGF-treated RAW264.7 cultured at 50 ng/ml for 48 h was another control to determine whether exogenous recombinant hVEGF_165_ could replace the endogenous protein and have a similar effect. qRT-PCR analysis indicated that in hVEGF_165_-ZsGreen1-RAW264.7 cells compared with other groups, the expression of endothelial marker genes, such as FLK-1, vWF, eNOS, VE-cadherin and Tie-2, were dramatically increased by 11-fold, 48-fold, 13-fold, 10-fold, and 13-fold, respectively (all *P*<0.01) (Figure 1A). There was no difference amongst other groups. Western blot measurement confirmed that corresponding significantly higher protein expression of FLK-1, vWF, and eNOS in hVEGF_165_-ZsGreen1-RAW264.7 than those in other groups (all *P*<0.01) (Figure 1B). There was no difference amongst other groups.

### Stable overexpression of VEGF induces macrophages to acquire functional characteristics of ELCs

**Figure 2:**
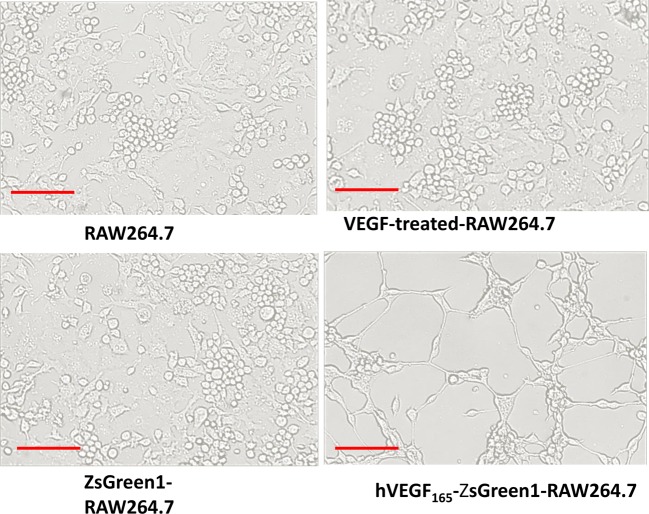
Effect of stable overexpression of VEGF on macrophages in tubular networks formation After 24 h of culture in Matrigel, hVEGF_165_-ZsGreen1-RAW264.7 formed tubular structures. But no similar structure was detected in untransfected RAW264.7, ZsGreen1-RAW264.7, or exogenous VEGF-treated RAW264.7. Scale bars =100 μm. All images are representatives of three independent experiments.

In order to further elucidate the functional role of stable overexpression of VEGF on the angiogenic potential of macrophages, the cells were cultured in Matrigel and tube formation was investigated *in vitro*. hVEGF_165_-ZsGreen1-RAW264.7 cells formed several obvious tubular-like structures after 24 h of culture in Matrigel. In contrast, no tubular structure was detected in untransfected RAW264.7, ZsGreen1-RAW264.7, and exogenous VEGF-treated RAW264.7 cells (Figure 2).

**Figure 3:**
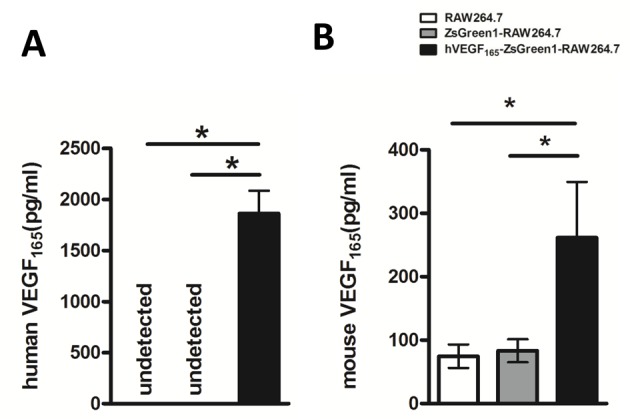
VEGF_165_ secretion level in supernatant after 72 h serum-free culture by ELISA (**A**) hVEGF_165_ production increased dramatically in hVEGF_165_-ZsGreen1-RAW264.7, but could not be detected in untransfected RAW264.7 and control transfected ZsGreen1-RAW264.7 (*P*<0.01). (**B**) Mouse VEGF_165_ secretion level in VEGF_165_-ZsGreen1-RAW264.7 is much higher than that in untransfected and control transfected groups. Data represent mean ± S.D. of three independent experiments each performed in triplicate. * indicates *P*<0.01.

*In vitro* hVEGF_165_ and mouse VEGF_165_ production, hVEGF_165_-ZsGreen1-RAW264.7 cells VEGF levels in the culture medium measured by ELISA showed that hVEGF_165_-ZsGreen1-RAW264.7 cells produced hVEGF 1861 ± 91 pg/ml. In contrast, it was not detectable in ZsGreen1-RAW264.7 or untransfected RAW264.7 cells (both *P*<0.01) (Figure 3A). Meanwhile, mouse VEGF_165_ production in the culture medium from hVEGF_165_-ZsGreen1-RAW264.7 cells was 262 ± 36 pg /ml. It is approximately three-fold higher than untransfected RAW264.7 and ZsGreen1-RAW264.7 (both *P*<0.01) (Figure 3B).

### Stable overexpression of VEGF reduces macrophage foam cell formation

**Figure 4:**
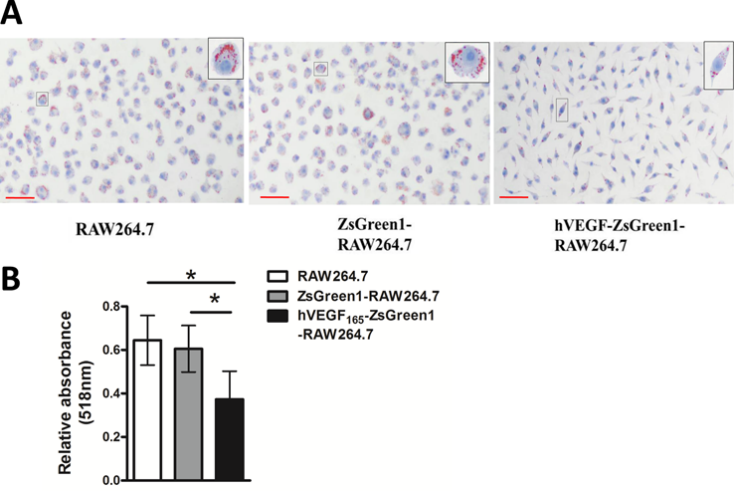
Foam cell formation assay (**A**) Representative images of foam cell formation assay. Untransfected RAW264.7, ZsGreen1-RAW264.7, and hVEGF_165_-ZsGreen1-RAW264.7 were treated with 100 μg/ml ox-LDL for 24 h. After fixation and staining with Oil Red O, the cells were observed by light microscopy. Cytoplasmic lipid droplets accumulated in RAW264.7 or ZsGreen1-RAW264.7, but hVEGF165-ZsGreen1-RAW264.7 cells showed significantly decreased lipid accumulation. Scale bars =50 μm. (**B**) Quantitative analysis of lipid accumulation in cells. The cells stained with Oil Red O were treated with 1 ml 60% isopropanol for 1 h to redissolve the oil red O and absorbance was detected at 518 nm through a spectrophotometer. The absorbance of oil red O was significantly decreased in hVEGF_165_-ZsGreen1-RAW264.7 compared with RAW264.7 or ZsGreen1-RAW264.7 cells. Data represent mean ± S.D. of three independent experiments each performed in triplicate. * indicates* P*<0.01.

We detected the capability of VEGF-modified macrophages to become foam cells. Incubation of untransfected RAW264.7 or ZsGreen1-RAW264.7 with 100 μg/ml ox-LDL for 24 h led to abundant cytoplasmic lipid droplets accumulation that was detected by Oil Red O staining. hVEGF_165_-ZsGreen1-RAW264.7 cells had a little lipid accumulation (Figure 4A).

Accordingly, the quantitation of lipid content was significantly decreased in hVEGF_165_-ZsGreen1-RAW264.7 cells compared with untransfected RAW264.7 or ZsGreen1-RAW264.7 cells (both *P*<0.01) (Figure 4B). Note that hVEGF_165_-ZsGreen1-RAW264.7 cells showed significantly decreased cytoplasmic lipid accumulation, indicating that stable overexpression of hVEGF suppressed macrophage foam cell formation.

### Stable overexpression of VEGF down-regulates expression of CD36 in macrophages

**Figure 5:**
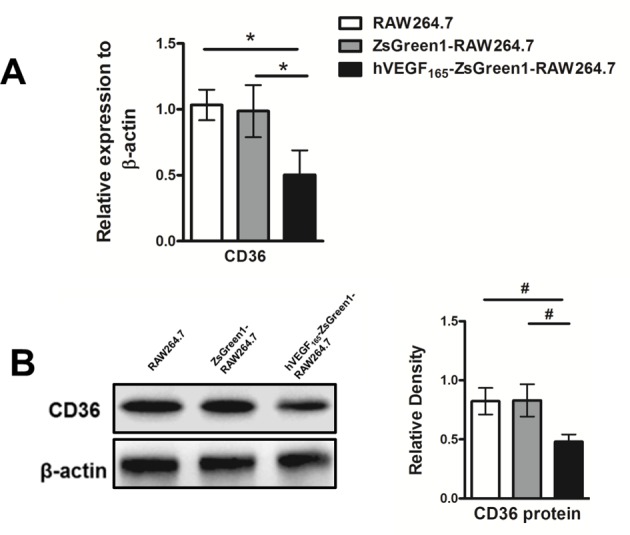
Stable overexpression of VEGF down-regulates expression of CD36 in macrophages (**A**) Quantitative analysis of mRNA expression of CD36 by qRT-PCR. After treating with 100 μg/ml ox-LDL for 24 h, mRNA expression of CD36 were significantly decreased in hVEGF_165_-ZsGreen1-RAW264.7 cells compared with that in RAW264.7 and ZsGreen1-RAW264.7. (**B**) Western blot analysis for endothelial cell markers expression. Protein expression of CD36 was significantly decreased in hVEGF_165_-ZsGreen1-RAW264.7 cells compared with RAW264.7 and ZsGreen1-RAW264.7. Data represent mean ± S.D. of three independent experiments each performed in triplicate. ^#^indicates *P*<0.05.

Therefore, after revealing that stable overexpression of VEGF inhibited lipid droplets accumulation in macrophages, we explored whether this effect was dependent on influx of lipids by analyzing the expression of CD36. As shown in Figure 5, after treating with 100 μg/ml ox-LDL for 24 h, *CD36* mRNA (Figure 5A) and protein expression (Figure 5B) were visibly reduced in hVEGF_165_-ZsGreen1-RAW264.7 cells compared with untransfected RAW264.7 or transfected control ZsGreen1-RAW264.7 cells (both *P*<0.05).

## Discussion

Macrophages are crucially involved in the whole process of atherosclerosis from early atherogenesis to advanced plaque progression [14,19,20]. During the process of initiation and formation of an atherosclerotic plaque, the inflammatory signals lead to monocyte recruitment into the damaged intima, where they differentiate into macrophages and internalize native and modified lipoproteins, resulting in foam cell formation. Moreover, foam cells can contribute further to, and thus amplify, lipoprotein modifications and retention. During the process of advanced plaques, macrophages can contribute to vulnerable plaque formation through the secretion of cytokines, proteases, and procoagulant/thrombotic factors [19]. During atherosclerotic complications healing (e.g. cardiac repair), monocytes might promote myofibroblast accumulation, angiogenesis, and myocardial healing and remodeling, thus show a protagonist or antagonist influence in post-acute coronary syndrome recovery [14]. Given that macrophages are widely involved and play an important role in the whole process of atherosclerosis, it can be taken as a potential future therapeutic target in atherosclerosis. Our hypothesis is that modified macrophages have new properties that can promote repair of damaged EC by acting as EPCs in early atherosclerosis and can inhibit foam cell formation during the progression of atherosclerosis.

Diversity and plasticity are the hallmarks of cells of the monocyte-macrophage lineage [13]. Monocytes are characterized by an extremely high developmental plasticity, under experimental conditions being able to differentiate into many kinds of cells ranging from epithelial cells, cartilage cells to fibroblasts, cardiomyocytes and neuronal cells, including endothelial lineage cells [14,21–24]. On the basis of our previous studies, we found the mouse macrophages transiently transfected with hVEGF_165_ might have a promising role like EPCs for cell-based therapy [15,16] and we have successfully established hVEGF_165_-ZsGreen1-RAW264.7 cells – a mouse modified macrophage cell line stably overexpressing hVEGF_165_ by lentiviral vector [17]. ZsGreen1 is a bright GFP, which can be used for tracking cells in animal experiment in the future. In the present study, we detected whether the hVEGF_165_-ZsGreen1-RAW264.7 cells can transdifferentiate into ELCs *in vitro.* The phenotypic features and function for identification of ELCs are similar to those used by Hu et al. [25]. qRT-PCR and Western blot analysis showed that levels of expression of classic endothelial markers were up-regulated in hVEGF_165_-ZsGreen1-RAW264.7 cells. The Matrigel assay further supported the notion that hVEGF_165_-ZsGreen1-RAW264.7 cells exhibited the characteristics of angiogenesis. Together, these findings displayed that VEGF-modified macrophage cells could transdifferentiate into ELCs *in vitro.*

The potential of macrophages to transdifferentiate into ELCs could be greatly beneficial for vascular repair. The use of hVEGF_165_-ZsGreen1-RAW264.7 cells might overcome limitations in cell numbers reported in tissue engineering and cell-base therapy using EPCs.

Autocrine VEGF may help to unravel the mechanism of transdifferentiation of macrophages to ELCs. In the present study, the RAW264.7 cells treated with exogenous recombinant hVEGF_165_ protein did not either increase the mRNA expression or the protein levels of endothelial markers differed. Also, no tubular structure was detected in Matrigel assay. Otherwise, RAW264.7 cells cultured with exogenous recombinant mouse VEGF_165_ protein or supernatant that was harvested from hVEGF_165_-ZsGreen1-RAW264.7 cells did not show morphological changes and significant changes in mRNA expression of endothelial markers eNOS and vWF (Supplementary Figure S2). These results suggested that addition of exogenous VEGF did not induce transdifferentiation of ELCs from macrophages. Lee et al. [26] reported that genetic deletion of *vegf* specifically in the endothelial lineage (VEGF^ECKO^) leads to progressive endothelial degeneration and addition of 100 ng/ml exogenous VEGF did not rescue the increased cell death exhibited by isolated VEGF^ECKO^ endothelial cells. Activation of the VEGFR2 in wild-type cells was suppressed by intracellular small molecule antagonists (SU4312) but not by extracellular blockade of VEGF (Avastin). Guangqi et al. [27] further demonstrated that endogenous VEGF-A forms a complex with VEGFR2 in endothelial cells and maintains a basal phosphorylation level of VEGFR2 as well as its downstream signaling proteins. This complex is localized within the early endosome antigen 1 (EEA1) endosomal compartment. In thie present study, stable transfection of macrophages with *hVEGF_165_* gene produced endogenous hVEGF protein by autocrine. In addition, ELISA showed that mouse VEGF production in the culture medium from hVEGF_165_-ZsGreen1-RAW264.7 cells was approximately three-fold higher than ZsGreen1-RAW264.7 or untransfected RAW264.7 cells, which suggested that stable overexpression of hVEGF_165_ in RAW264.7 promoted increased autocrine mouse VEGF_165._ Both endogenous hVEGF_165_ and mouse VEGF_165_ may contribute to maintaining the transdifferentiated ELCs homeostasis and phenotype.

In view of this, macrophages are the one of the major resources of the lipid-laden foam cells in atherosclerosis, the foam cell formation assay is used as a biological indicator of the therapeutic effect of an anti-atherogenic treatment [28]. Our results demonstrated that the stable overexpression of VEGF decreased the number of intracellular lipid droplets and total lipid content in RAW 264.7 macrophages, and inhibited ox-LDL induced foam cell formation.

To further attempt to unveil the possible mechanism, we measured the mRNA abundance and protein levels of CD36 on macrophages to see whether endogenous VEGF inhibit foam cell formation via reduced cholesterol influx. CD36 is one of scavenger receptors responsible for macrophages uptake of ox-LDL [29] and accounts for approximately 60–70% of macrophage-derived foam cell formation [30]. Yao et al. [30] offered a new mechanism to explain the macrophage uptake of ox-LDL without limitation by demonstrating that CD36-mediated ox-LDL uptake in macrophages triggered endoplasmic reticulum (ER) stress response, which, in turn, up-regulated CD36 mainly at the protein level, enhancing the foam cell formation by uptaking more ox-LDL [30].

Our results in the present study indicated that *CD36* mRNA and protein expression as well as lipid-droplet accumulation were attenuated in hVEGF_165_-ZsGreen1-RAW264.7 cells, suggesting that down-regulation of CD36 expression in VEGF-modified macrophages is probably one of the mechanisms of reduction in foam cell formation. We also detected the gene and protein expression of ATP-binding cassette transporter A1 (ABCA1), a critical regulator of lipid efflux from cells; however, there was no significant difference amongst the four groups (results not shown).

Our results raised the prospect that macrophages stably overexpressing VEGF may attenuate the progression of atherosclerosis by reducing foam cell formation, as well as inhibit the initiation of atherosclerosis by repairing the injured arterial endothelial cells in early stage. These *in vitro* data provided a solid basis for further *in vivo* investigation of atheroprotective effect of hVEGF_165_-ZsGreen1-RAW264.7 cells as a cell-based therapy.

## Abbreviations

ELC: endothelial-like cell
eNOS: endothelial NO synthase
EC: endothelial cell
EPC: endothelial progenitor cell
FLK-1: fetal liver kinase 1
hVEGF_165_: human vascular endothelial growth factor 165
ox-LDL: oxidized low density lipoprotein
TBS: Tris buffered saline
TMB: tetramethylbenzidine
VEGF: vascular endothelial growth factor
VEGFR2: vascular endothelial growth factor receptor 2
VE-cadherin: vascular endothelial-cadherin
vWF: von Willebrand factor
